# Sestrin2 reduces cancer stemness via Wnt/β-catenin signaling in colorectal cancer

**DOI:** 10.1186/s12935-022-02498-x

**Published:** 2022-02-11

**Authors:** Jinlai Wei, Xiangru Zheng, Wenjun Li, Xiaoli Li, Zhongxue Fu

**Affiliations:** 1 Department of Gastrointestinal Surgery, The First Affiliated Hospital of Chongqing Medical University, Chongqing, 400016 China; 2 The Third Affiliated Hospital of Chongqing Medical University, Chongqing, China; 3 College of Pharmacy, Chongqing Medical University, Chongqing, China

**Keywords:** Sestrin2, Colorectal cancer, Wnt/β-catenin, Cancer stemness, Therapeutic target

## Abstract

**Background:**

Colorectal cancer (CRC) is one of the most commonly diagnosed cancers in both men and women in China. In previous studies, Sestrin2 was demonstrated to have functions in CRC. However, the relationship between Sestrin2 and cancer stemness has not been reported.

**Methods and results:**

To investigate the contribution of Sestrin2 in CRC, we performed bioinformatics analysis of The Cancer Genome Atlas datasets and found that Sestrin2 was downregulated in CRC. Using a lentivirus vector, we verified that Sestrin2 suppressed CRC cell proliferation, migration, and colony formation. Furthermore, sphere formation, flow cytometry, quantitative PCR, and western blot analysis verified the influence of Sestrin2 on cancer stemness, including the expression of cluster of differentiation 44, octamer-binding transcription factor 4, sex-determining region Y-Box 2, CXC chemokine receptor 4, and the Wnt pathway downstream factors β-catenin and c-Myc. Consistently, the Wnt pathway activator BML-284 partially rescued the effects of Sestrin2 on the expression of proteins related to cancer stemness. Furthermore, in a mouse xenoplant model, tumors expressing Sestrin2 were significantly reduced in size with corresponding changes in cancer stemness.

**Conclusions:**

Collectively, our results suggest that Sestrin2 inhibits CRC cell progression by downregulating the Wnt signaling pathway. Thus, Sestrin2 may be a promising therapeutic target for CRC.

**Supplementary Information:**

The online version contains supplementary material available at 10.1186/s12935-022-02498-x.

## Background

Colorectal cancer (CRC) is one of the most commonly diagnosed cancers in both men and women in China [1]. Standard anticancer therapies for CRC include surgery and chemotherapy, but the survival rate is still unsatisfactory. The failure of standard anticancer treatments might be due to cancer stem cells (CSCs) [2]. CSCs are tumor cells that have stemness properties, namely, self-renewal, tumor initiation capacity, and long-term repopulation potential [3]. In addition to CSCs, some cancer cells also have stemness properties, and these cells might be a result of the transformation of typical stem cells in the tissue or emerge from nonstem cell regulation due to exposure to stemness factors [4]. Targeting CSCs would be a promising therapeutic method. Therefore, investigating CSCs to find new molecules and mechanisms in CRC would reveal novel therapeutic targets.

Sestrin family members, including Sestrin1, Sestrin2, and Sestrin3, can be induced by cellular stresses, such as ER stress, DNA damage and ROS accumulation [5–7]. In the Sestrin family, Sestrin2 has shown regulatory activities in some cancers [8]. Knocking down Sestrin2 in non-small cell lung cancer cells suppressed lung cancer progression [9]. Downregulation of Sestrin2 promoted cell proliferation, migration, and ROS production in endometrial cancer cells [10]. In CRC, our previous study demonstrated reduced expression of Sestrin2 relative to normal colorectal epithelial tissues [11]. Other studies have shown that Sestrin2 has many other functions in CRC; for example, Sestrin2 is involved in inducing the apoptotic process of HCT116 CRC cells [12], and its overexpression inhibits the migration, invasion, and growth of CRC [13]. However, the relationship between Sestrin2 and CRC stemness has not been previously reported.

This study aimed to investigate the role of sestrin2 in the cancer stemness of CRC. First, the effects of upregulating Sestrin2 on the survival, migration, and colony formation of CRC cells were studied, and the expression of cancer stemness in CRC cells with high Sestrin2 expression was detected. Second, the relationship between Sestrin2 and Wnt signaling was explored. Finally, we observed that upregulation of Sestrin2 inhibited tumor growth in CRC cells in vivo. Our results suggest that Sestrin2 inhibits CRC cell progression by downregulating the Wnt signaling pathway.

## Materials and methods

### Cell culture

The human CRC cell lines were purchased from Shanghai Zhong Qiao Xin Zhou Biotechnology Co., Ltd. (Shanghai, China). HCT-116 cells were cultured in McCoy’s 5A medium (Biological Industries, Beit HaEmek, Israel), and SW620 cells were cultured in RPMI 1640 medium (Biological Industries) supplemented with 10% fetal bovine serum (FBS) (Biological Industries). Cells were cultured at 37 °C with 5% CO_2_ in humidified incubators.

### Infection with lentiviral constructs

Lentiviral constructs expressing Sestrin2 (LV-Sestrin2) and a nontargeted green fluorescence protein (GFP) virus (LV-GFP) were purchased from GeneChem (Shanghai, China). Cells (1.5 × 10^4^) were plated in 24-well plates on the day before transfection. Lentiviral constructs were transduced at a multiplicity of infection of 10 in HCT-116 and SW620 cells using HiTransG P transfection reagent (GeneChem). The infected cells were maintained in fresh medium without FBS for 12 h and then washed with phosphate-buffered saline (PBS), after which the medium was replaced with fresh medium supplemented with 10% FBS. Puromycin (2 μg/mL; Beyotime, Beijing, China) was used to generate stable expression cell lines in HCT116 and SW620 cells.

### Quantitative PCR

The RNAsimple Total RNA Kit (TIANGEN, Beijing, China) was used to extract total RNA according to the manufacturer’s instructions. All-in-One cDNA Synthesis SuperMix (Bimake, USA) was used to generate first-strand cDNA. The expression level of the cDNAs was normalized to that of β-actin by the comparative CT method. The primer sequences used in this study are provided in Supplementary Table 1.

### Cell proliferation assay

A total of 500 cells per well were plated in the wells of a 96-well plate. Cell viability was measured using Cell Counting Kit-8 (CCK-8) (Bimake) and the Thermo Scientific™ Varioskan™ LUX Multimode microplate reader (Thermo Fisher Scientific, Waltham, MA, USA) at 450 nm once a day for 7 days.

### Extreme limiting dilution assay in vitro

The procedure of the extreme limiting dilution assay (ELDA) was the same as that described previously [14]. Briefly, cells were seeded in U-bottom 96-well plates at final cell concentrations of 1000, 100, 10, and 1 per well with 200 µL SCM. After 2 weeks, the wells containing cell spheres were counted, and empty wells were excluded. The frequency of CSCs was calculated on the website (http://bioinf.wehi.edu.au/software/elda/) [15].

### Sphere formation

Sphere cells were induced from adherent cells for 2 weeks in stem cell medium (SCM), which is a DMEM/F-12 medium supplemented with epidermal growth factor (20 ng/mL, MedChemExpress, MCE), basic fibroblast growth factor (20 ng/mL, MCE), and B-27 (2%; Invitrogen, Carlsbad, CA, USA). A total of 200 sphere cells were plated into each 96-well plate with 200 µL SCM. The number of spheres was counted after 2 weeks. For the second sphere assay, sphere cells were separated using ACCUTASE (YEASEN). A total of 200 cells were plated per well in each 96-well plate with 200 µL SCM. The number of spheres was counted after 2 weeks.

### Soft agar colony formation assay

To evaluate the anchorage-independent growth of LV-Sestrin2 cell lines, a concentration of 1 × 10^4^ cells was suspended in SCM containing 0.36% agar and poured on an agar bed (SCM with 0.75% agar). After 3 weeks, the sphere number was counted after 0.04% crystal violet staining.

### Wound healing assay

HCT116 cells and SW620 cells (4 × 10^5^ cell/well) were seeded into 6-well plates. On the next day, straight lines were scratched on the cell monolayer using a 200 µL pipette tip. After washing away the cell debris with PBS, fresh culture medium with 2% FBS was added. Images of the migratory cells were captured at 0 h, 24 h and 48 h. The relative cell migration rate of each group (initial area minus 48 h scratch area) was normalized against the scratched area at 0 h (ImageJ software 1.52a).

### Transwell assay

A total of 1 × 10^6^ cells were added to the upper chamber (0.8 µM in 6-well plates) of a Transwell chamber (JET, China) in serum-free culture medium. The lower chamber contained medium supplemented with 10% FBS. After 24 h of culture, the cells on the upper side of the filter membrane were removed. The rest of the cells were fixed with 5% paraformaldehyde for 20 min, dyed using Crystal Violet Staining Solution (Beyotime, China), photographed at 200 × and counted in five random fields.

### Flow cytometry

Cells were detached into single cells, washed with cold PBS, preincubated with Human TruStain FcX™ (422,301; BioLegend, San Diego, CA, USA), and then incubated with CD44-APC (B265921; BioLegend) on ice in the dark. The cells were assayed by flow cytometry.

### Wnt/β-catenin pathway inhibition assay

HCT-116 cells in the LV-Sestrin2 and LV-GFP groups were treated with 0.5 µM BML-284 (Wnt signaling activator; MedChemExpress, Monmouth Junction, NJ, USA) in dimethyl sulfoxide for 24 h and then collected for western blot analysis. In the sphere formation assay, 0.1 µM BML-284 was mixed with the cells and then they were plated in 96-well plates with SCM. The number of spheres was counted after 2 weeks.

### Western blot analysis

Cells were washed with PBS and lysed in RIPA buffer with Protease Inhibitor Cocktail (Bimake) on ice. Cell lysates were centrifuged (12,000 rpm) at 4 °C for 20 min and then quantified using the BCA Protein Assay Kit (Beyotime). The lysate was denatured with sodium dodecyl sulfate–polyacrylamide gel electrophoresis (SDS–PAGE) sample loading buffer (Beyotime), followed by SDS–PAGE and electrotransfer to polyvinylidene difluoride membranes (Millipore, Darmstadt, Germany). The membranes were incubated overnight at 4 °C with anti-Sestrin2 (Proteintech Group 10795-1-AP 1:1000), anti-CD44 (Proteintech Group 15675-1-1AP 1:1000), anti-Sox2 (Proteintech Group 11064-1-AP 1:1000), anti-Oct4 (Proteintech Group 11263-1-AP 1:1000), anti-CXC chemokine receptor 4 (Cxcr4) (Proteintech Group 11073-2-AP 1:1000), anti-β-catenin (Proteintech Group 51067-2-AP 1:1000) and anti-β-actin (Proteintech Group 20536-1-AP 1:1000) at the appropriate dilution and then incubated with the secondary antibody at room temperature for 2 h. The bands were visualized using BeyoECL Plus (Beyotime).

### Xenograft mouse model

A xenograft mouse model was established by subcutaneous injection. One million HCT-116 cells in the LV-Sestrin2 and LV-GFP groups were injected into the hips of 6-week-old female BALB/c-nu mice (Beijing Vitalstar Biotechnology, China). All animal studies were approved by the Ethics Committee of Chongqing Medical University. The tumor size was measured 5 days later and then every 3 days. The tumor volume (V = l × w^2^/2) was calculated by measuring the length (l) and width (w). Mice were euthanized in a CO_2_ chamber 20 days after cell injection.

### Immunohistochemistry (IHC)

IHC assays were conducted as reported previously [16]. Antibody against Sestrin2 (Proteintech Group 10795-1-AP 1:500), Ki-67 (Proteintech Group, 27309-1-AP, 1:500) or β-catenin (Proteintech Group 51067-2-AP 1:500) were incubated at room temperature for 2 h. The biotinylated goat anti-rabbit antibody (Beyotime China, A0279, 1:200) was used as the secondary antibody. The diaminobenzidine tetrachloride (Beyotime China, P0203) was used for staining at room temperature for 1 min.

### Bioinformatics analysis

The colorectal cancer dataset comprised mRNA-seq data from TCGA tumors (https://tcga-data.nci.nih.gov/tcga/). The two-gene correlation map was generated by the R software package ggstatsplot, and the multigene correlation map was displayed by the R software package heatmap. We used Spearman’s correlation analysis to describe the correlation between the quantitative variables without a normal distribution. A *P* value of less than 0.05 was considered statistically significant.

### Statistical analysis

Except where otherwise noted, the experiments were repeated at least three times. All statistical analyses were performed using GraphPad Prism Version 8. The Mann–Whitney test or T test was used to evaluate the significance of the differences between two groups of data. *P* ≤ 0.05 was considered statistically significant. Medians were used to summarize the data.

## Results

### Sestrin2 inhibits CRC cell proliferation, migration, and colony formation

**Fig. 1:**
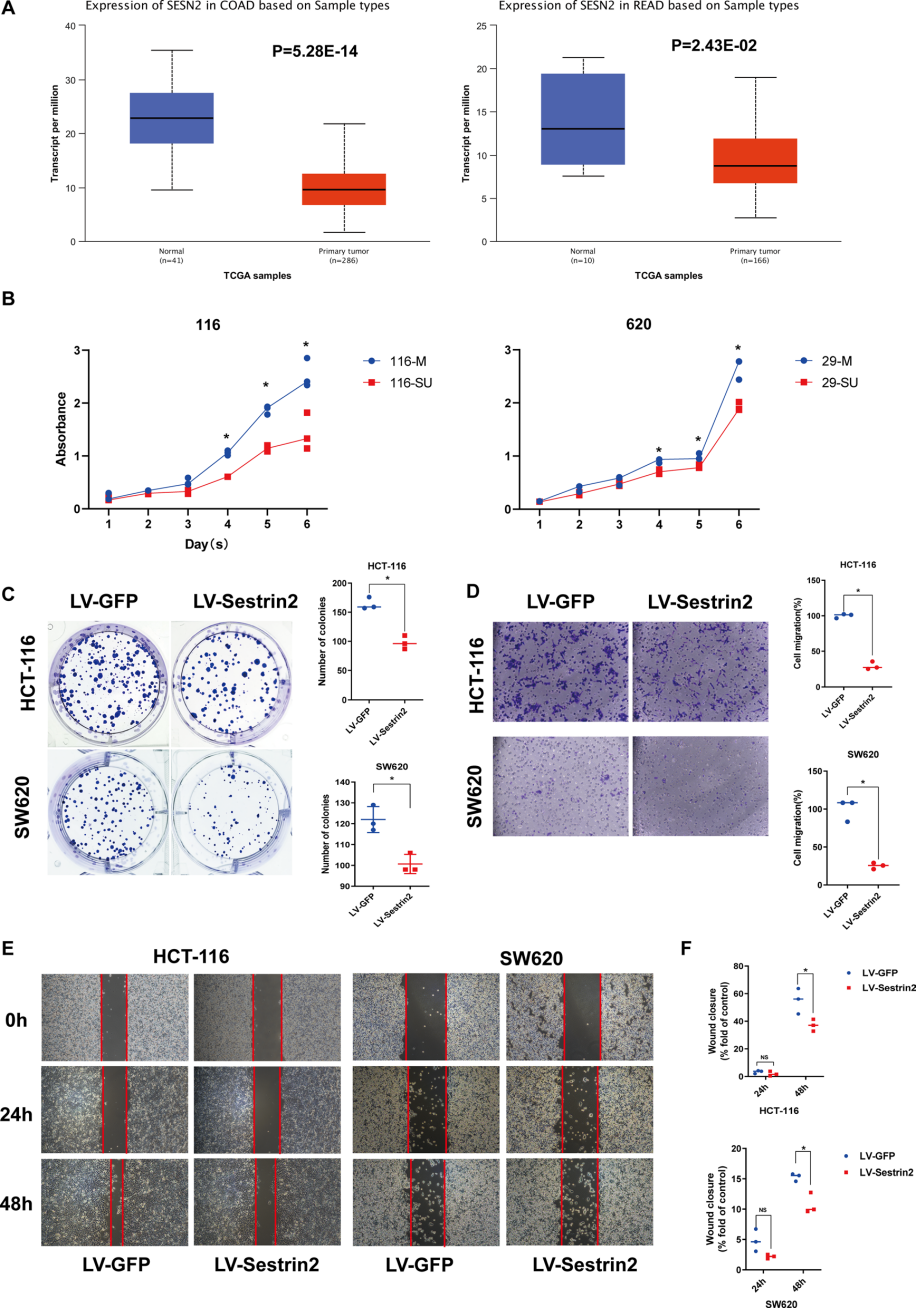
Sestrin2 has anticancer effects on CRC cells. **A** Sestrin2 was downregulated in both COAD (*P* = 0.28 × 10^–14^) and READ (*P* = 0.024) compared to normal tissue in TCGA.** B** Cell proliferation was markedly inhibited after Sestrin2 upregulation, as determined by the CCK-8 assay. (*P = 0.05; Mann–Whitney test; connected by medians) **C** The colony formation assay showed that the number of colonies formed by LV-Sestrin2-infected cells was smaller than that of LV-GFP-infected cells. The right panel shows the number of colonies (*P = 0.05; Mann–Whitney test; lines showed medians). **D** Cell migration was detected by Transwell assays. The cell number was counted by ImageJ and normalized to the LV-GFP group (right panel) *P = 0.05; Mann–Whitney test; lines showed medians). **E** Cell migration was measured by the wound healing assay. The area of migration was quantified as the mean ± standard deviation (SD) and normalized to the respective LV-GFP group (right panel); *P = 0.05; Mann–Whitney test; lines showed medians

To evaluate the effects of Sestrin2 on CRC, we first investigated the mRNA expression of Sestrin2 compared to the corresponding adjacent tissues in The Cancer Genome Atlas datasets using the UALCAN website (UALCAN: http://ualcan.path.uab.edu) (Fig. 1A) [17]. The results showed that Sestrin2 was significantly downregulated in both colon adenocarcinoma (COAD) and rectum adenocarcinoma (READ).

To investigate the effects of Sestrin2 on cancer phenotypes in vitro, we introduced LV-Sestrin2 and LV-GFP lentivirus particles into HCT116 and SW620 cells, and proliferation, migration, and colony formation were evaluated. To detect the effect of Sestrin2 on CRC proliferation, a CCK-8 assay was performed, and the results showed that the absorbance of the LV-Sestrin2 group began to differ significantly from that of the LV-GFP group after 3 days and 4 days in the HCT-116 and SW620 cells, respectively (Fig. 1B). These results suggested that Sestrin2 inhibited the proliferation of CRC cells.

In addition, the colony formation assay showed that the number of colonies formed was decreased from 164.0 ± 10.44 in LV-GFP to 97.67 ± 11.59 in LV-Sestrin2 in HCT-116 cells and from 122.0 ± 6.245 to 100.7 ± 4.619 in SW620 cells (Fig. 1C).

Furthermore, to investigate the migration ability, Transwell and wound healing assays were conducted. The Transwell assay results showed that the migration of LV-Sestrin2-transduced CRC cells was inhibited (Fig. 1D). The wound healing assay also showed that HCT116 and SW620 cells healed less than 60% and more than 30% of the area, respectively, in the LV-GFP group; however, the healing ability was reduced to less than 20% and nearly 10% after LV-Sestein2 transduction in these two cell lines (Fig. 1E). A similar trend was also observed in the invasion assay (Additional file 1: Figure S1). The data suggested that Sestrin2 inhibited migration and invasion in CRC. These results demonstrate that Sestrin2 is downregulated in CRC patients and has antiproliferative and antimigratory effects on CRC cells.

### Sestrin2 inhibits the cancer stemness in CRC cells

**Fig. 2:**
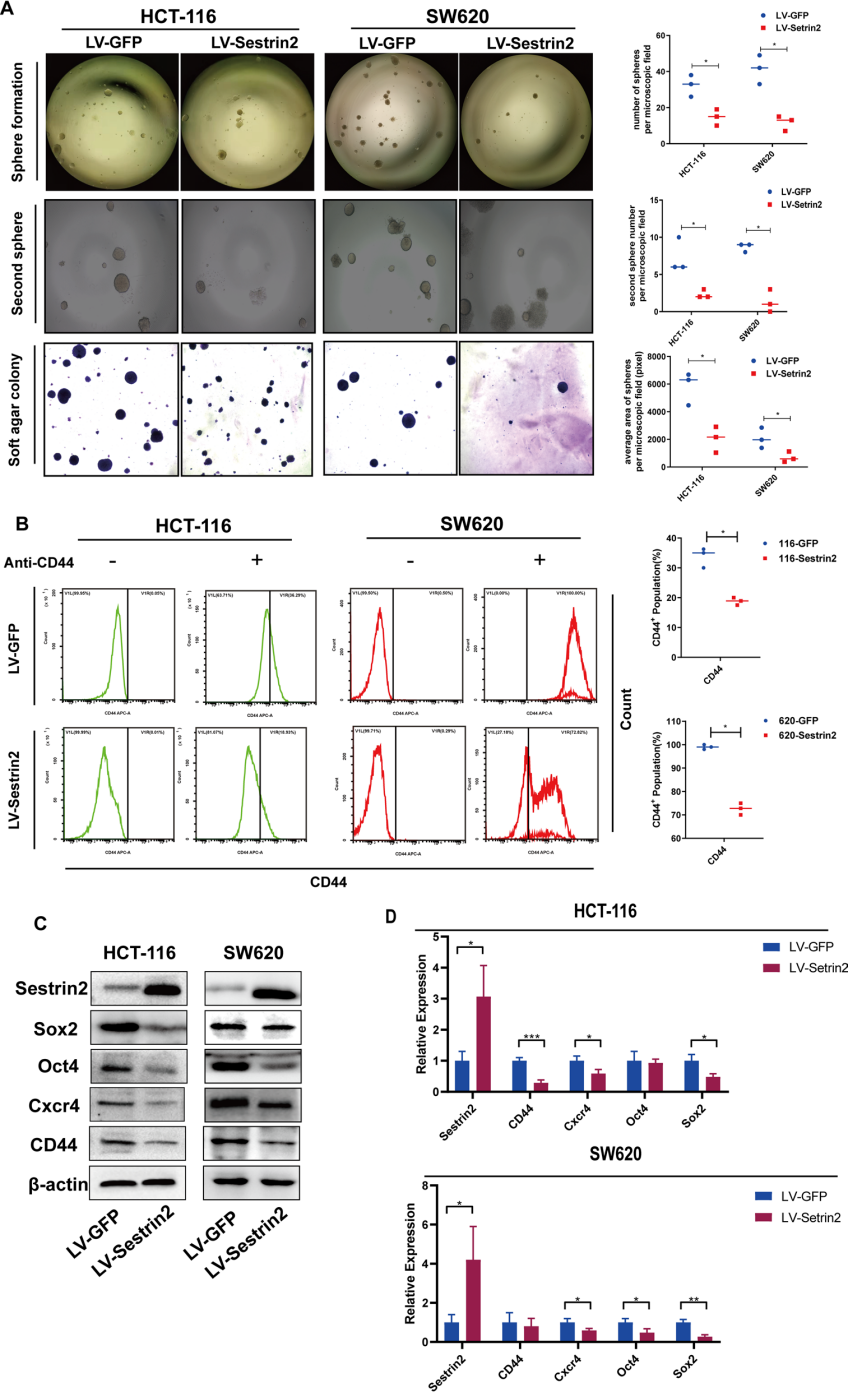
Sestrin2 inhibits cancer stemness in CRC cells. **A** The cell sphere formation assay (top), second cell sphere formation (middle) and soft agar colony formation assay (bottom) showed that the number of spheres and the average area of the soft agar colonies were both decreased; *P = 0.05; Mann–Whitney test; lines showed medians. **B** Flow cytometry was used to detect the percentages of CD44+ cells. The percentages of CD44+ cells in the LV-GFP group were larger than those in the LV-Sestrin2 group (*P = 0.05; Mann–Whitney test; lines showed medians). **C** Representative western blot images of the effect of LV-Sestrin2 on the expression levels of Sestrin2, Sox2, Oct4, Cxcr4, and CD44. β-actin was used as a loading control. **D** The relative mRNA expression of Sestrin2, Sox2, Oct4, Cxcr4, and CD44 in LV-Sestrin2 CRC cells normalized to the LV-GFP group (*P < 0.05, **P < 0.01, ***P < 0.001, T test)

CSCs play a significant role in cancer progression, such as tumor growth, recurrence, and metastasis [18]. Therefore, we hypothesized that Sestrin2 affects CSCs in CRC cells. Self-renewal and tumor initiation are basic characteristics of CSCs [19]. To identify the effect of Sestrin2 on self-renewal of CRC adhering cells, a sphere assay was used. The sphere assay results showed that in HCT-116 cells, the LV-GFP cells formed 32.33 ± 6.03 colonies, while LV-Sestrin2 formed 14.67 ± 4.51 colonies. The SW620 cell line had 41.33 ± 8.02 colonies in the LV-GFP group and 11.67 ± 4.16 colonies in the LV-Sestrin2 group (Fig. 2A). To investigate the effect of Sestrin2 on the self-renewal ability of CSCs, a second sphere formation assay was performed [20]. Unsurprisingly, the colony number formed in the second sphere formation assay had a similar trend to that of the first sphere formation assay. LV-GFP HCT-116 sphere cells formed 7.33 ± 2.31 colonies, LV-Sestrin2 formed 2.33 ± 0.58 colonies, and SW620 cells formed 8.67 ± 0.58 and 1.33 ± 1.53 colonies, respectively, suggesting that Sestrin2 inhibited CSC self-renewal in CRC (Fig. 2A).

The strength of anchorage-independent growth in soft agar assays reflects the tumorigenesis of the cells [21]. Soft agar assays are able to imitate an in vivo microenvironment [22]. HCT-116 and SW620 cells in the LV-GFP and LV-Sestrin2 groups were cultured in soft agar to form colonies over 3 weeks. The average colony sizes of the LV-GFP and LV-Sestrin2 HCT-116 colonies were 5816.67 ± 1190.17 and 2035.67 ± 941.48, respectively, while the average sizes of the SW620 cells were 2067.67 ± 739.55 and 697.33 ± 386.35 pixels, respectively (Fig. 2A). Moreover, to determine the number of colorectal cancer-initiating cells, we tested colony formation using limited dilutions of cells, and the results showed that LV-Sestrin2 reduced the CSCs in the HCT-116 and SW620 cell lines (Table 1). These data suggest that Sestrin2 decreased self-renewal and tumor formation ability in CRC.

**Table 1:** Limiting dilution data showing the effect of Sestrin2 expression on the frequency of CSCs

Cells seeded		1	10	100	1000	Estimated frequency of CSCs	*P* value
HCT-116	LV-GFP	62/78	152/162	89/92	24/24	1 in 6 cells	0.087
	LV-Sstrin2	78/105	136/170	86/91	24/24	1 in 7 cells	
SW620	LV-GFP	45/90	101/145	76/94	24/24	1 in 19 cells	4.24 × 10–8
	LV-Sstrin2	51/128	96/172	74/96	22/24	1 in 35 cells	

In addition, molecules related to cancer stemness were evaluated. Cell surface proteins are used as CSC markers for different types of tumors, for example, cluster of differentiation 34-positive (CD34+)/CD38 − for leukemia cells, CD13/CD45/CD90 for liver cancer, CD117/CD90/epithelial cell adhesion molecule for lung cancer [23, 24], and CD44 for colon and gastric cancers [25]. We used flow cytometry to investigate the expression strength of the stemness biomarker CD44 and the number of CD44+ cells. Flow cytometry showed that both the percentage of CD44+ cells and the expression strength of CD44 were decreased in Sestrin2-transfected CRC cells (Fig. 2B).

The stemness state is governed by stem cell factors such as the four Yamanaka factors sex-determining region Y-Box 2 (Sox2), octamer-binding transcription factor 4 (Oct4), Kruppel-like factor 4, and c-Myc [26]. Stemness-related markers, including CD44, Oct4, Sox2, and Cxcr4, were investigated by western blotting and qPRC to illustrate the effect of Sestrin2 on protein expression and mRNA transcription. The results showed that LV-Sestrin2 downregulated stemness-related markers at both the mRNA and protein levels in HCT-116 and SW620 cells (Fig. 2C–D, Additional file 2: Figure S2A).

Furthermore, whether Sestrin2 affected stemness in CSCs was investigated by western blot. Sphere cells were induced from adherent cells in SCM for 2 weeks, and then western blotting was conducted. The results showed similar protein expression trends in sphere cells, which hinted that Sestrin2 was able to reduce stemness in CSCs (Additional file 2: Figure S2B).

Epithelial-mesenchymal transition (EMT) promotes cancer stemness. We also investigated the expression of E-cadherin, an EMT negative correlation marker, in LV-GFP and LV-Sestrin2 cells by western blotting (Additional file 2: Figure S2C). The results showed that Sestrin2 increased the expression of E-cadherin, which indicated that Sesterin2 negatively regulated EMT and cancer stemness.

These data prove that Sestrin2 suppresses the expression of stemness-related markers and reduces cancer stemness in CRC cells.

### Sestrin2 reduces cancer stemness via the Wnt/β-catenin pathway

**Fig. 3:**
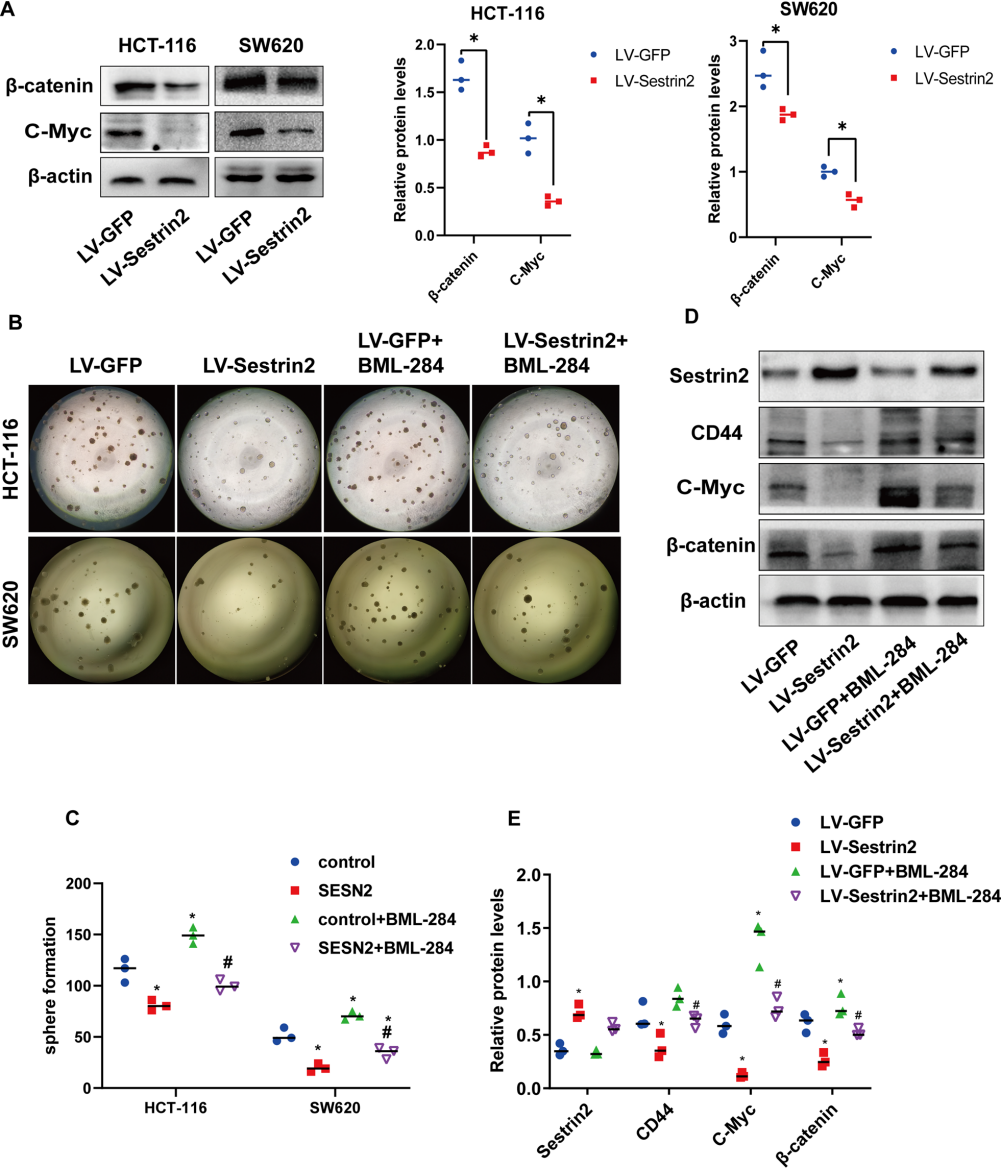
The effects of Sestrin2 on cancer stemness are mediated by the Wnt/β-catenin pathway. **A** Western blot analysis of Wnt/β-catenin signaling-related proteins. The relative protein expression is shown in the right panel. β-Actin was used as a loading control *P = 0.05; Mann–Whitney test; lines showed medians). **B** Sphersphere formation assay in LV-Sestrin2 and LV-GFP CRC cells with or without exposure to BML-284 (0.1 µM). **C** Number of spheres, * compared with LV-GFP, *P = 0.05; ^#^ compared with LV-Sestrin2, ^#^*P* = 0.05; Mann–Whitney test; lines showed medians. **D** Western blot analysis of proteins related to the Wnt/β-catenin pathway (β-catenin, c-Myc) and cancer stemness (CD44) in LV-Sestrin2 and LV-GFP CRC cells with or without incubation with BML-284 (0.5 µM) for 24 h in HCT-116 cells.** E** The relative protein expression. * Compared with LV-GFP, *P = 0.05, ^#^ compared with LV-Sestrin2, ^#^*P* = 0.05, Mann–Whitney test; lines showed medians

Wnt signaling is one of the key pathways that regulate development and stemness in cancer [27]. The function of the Wnt pathway in CSCs is commonly accepted and it depends on the amount of β-Catenin in the cytoplasm [28, 29]. β-catenin is able to activate not only proliferation and transmission factors, such as c-Myc, c-Jun, and CCND1 (the gene encoding cyclin D1) but also epidermal growth factor receptors, such as CD44 and CD133. Activation of the Wnt/β-Catenin pathway increases the number of CSCs by regulating self-renewal and homeostasis in cancer cells [30]. We observed the protein expression of β-catenin and the downstream target c-Myc. Our observations showed that the expression of β-catenin and c-Myc was downregulated (Fig. 3A). Furthermore, we used the Wnt/β-catenin pathway activator BML-284 to rescue the effects of Sestrin2 and found that BML-284 was able to partly rescue the effects of Sestrin2 using the sphere assay (Fig. 3B–C), consistent with the western blot analyses in HCT-116 cells (Fig. 3D–E). Our findings suggest that Sestrin2 regulates cancer stemness via the Wnt/β-catenin pathway.

In addition, reactive oxygen species (ROS) were reported to play an important role in maintaining cancer stemness [31]. Sestrin2 was able to suppress ROS in cancer cells [32]. To investigate whether sestrin2 affects cancer stemness by regulating ROS, scavengers of ROS, sodium pyruvate and carboxy-PTIO were used. The results showed that scavengers of ROS, although they slightly downregulated the protein expression of CD44, were unable to rescue the CD44 inhibition caused by Sestrin2 overexpression, regardless of whether the scavenger was used alone or in combination (Additional file 3: Figure S3). These data indicate that Sestrin2 does not regulate cancer stemness through ROS.

### Sestrin2 inhibits cancer stemness in vivo

**Fig. 4:**
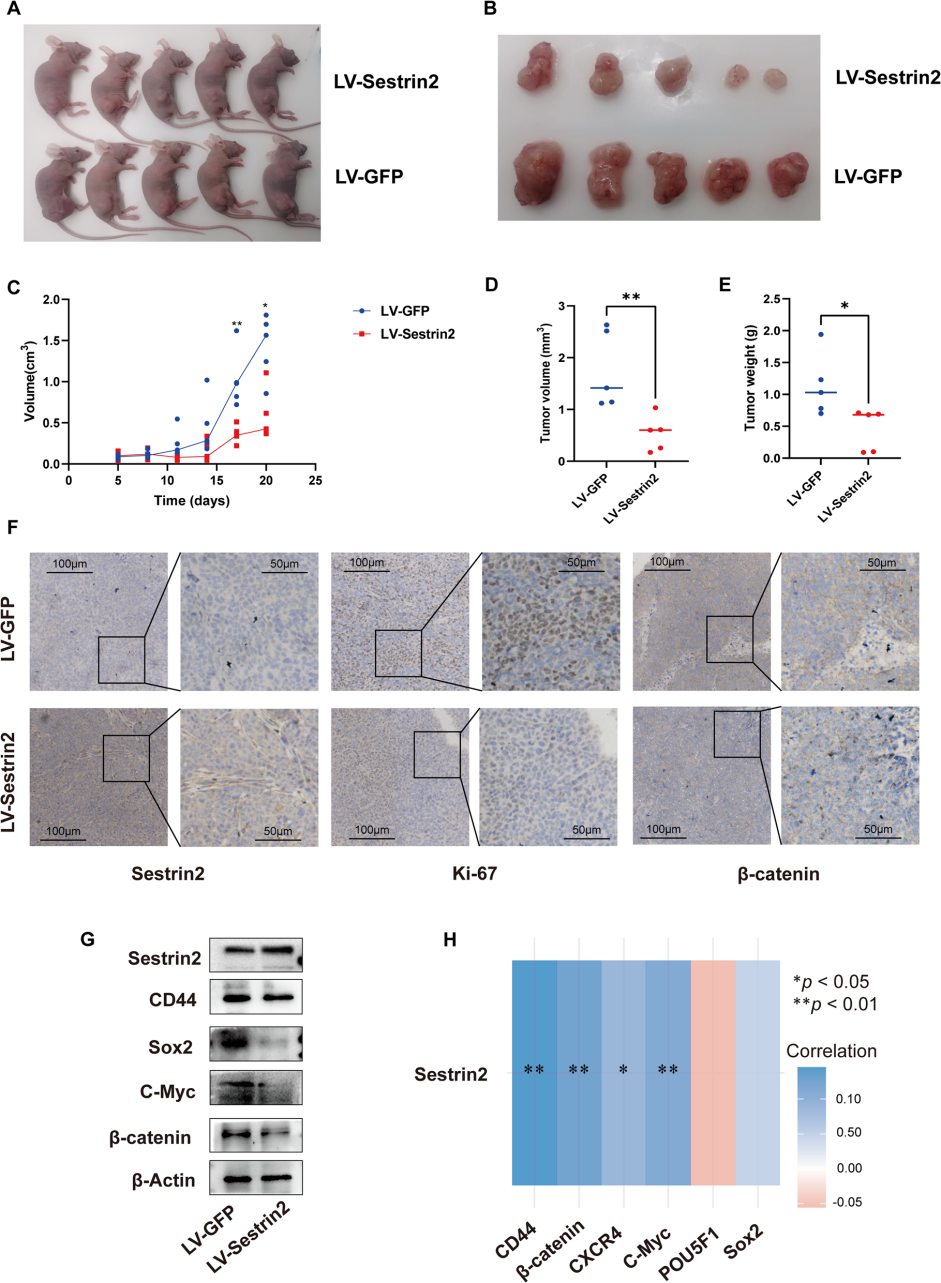
Sestrin2 inhibits cancer stemness in vivo. **A** LV-Sestrin2 and LV-GFP cells were transplanted into the hips of mice and after 3 weeks **B** The tumors were excised. **C** Tumor volume data (**P = 0.008, *P = 0.02, Mann–Whitney test; connected by medians). **D** The tumor volumes were measured after removal from the mice (**P = 0.008, Mann–Whitney test; lines showed medians). **E** The tumor weights were measured (*P = 0.02, Mann–Whitney test; lines showed medians). **F** HIC of Sestrin2, Ki-67 and β-catenin. **G** Western blots showing the protein expression of Sestrin2, CD44, Sox2, c-Myc, and β-catenin in mouse tumors. β-Actin was used as a loading control. **H** A heatmap of the correlations between CD44, β-catenin (CTNNB1), CXCR4, c-Myc (MYC), OCT4 (POU5F1), SOX2 and Sestrin2 (SESN2). Red represents a positive correlation, blue represents a negative correlation, and darker colors represent stronger correlations. Asterisks represent levels of significance (*P < 0.05, **P < 0.01, T test)

To evaluate the effects of Sestrin2 in vivo, a subcutaneous xenotransplant tumor model was used. A concentration of 1 × 10^7^ cells from the LV-Sestrin2 or LV-GFP groups was injected into each of five female BALB/c-nu mice. The xenotransplant tumors were observed 5 days later and then every 3 days. Twenty days after cell injection, the mice were euthanized, and the tumors were removed (Fig. 4A–B). The growth rate of the tumors produced by HCT116-Sestrin2 cells was significantly slower than that of tumors produced by HCT116-GFP cells, although the weights of the mice were not significantly different (Fig. 4C, Additional file 4: Figure S4A). After the tumors were removed, the volume and weight were measured again, and the tumors from the LV-Sestrin2 group were inhibited (*P* = 0.0101 in volume, *P* = 0.0334 in weight, Fig. 4D–E).

We then performed IHC of the cell proliferation markers Ki-67 and β-catenin in the tumor tissues to determine the effect of LV-Sestrin2 in vivo. As expected, the expression of Ki-67 and β-catenin in the LV-Sestrin2 group was lower than that in the LV-GFP group (Fig. 4F). Furthermore, we observed the CSC-related protein expression of xenotransplant tumors by western blot analysis. The results showed that upregulation of Sestrin2 inhibited the protein levels of Sox2, c-Myc, and β-catenin in xenotransplant tumors (Fig. 4G, Additional file 4: Figure S4B). The efficiency of tumor formation and cancer stemness was decreased by Sestrin2 in vivo.

Finally, to confirm our findings in CRC patients, the mRNA expression correlations between Sestrin2 and CD44, Oct4, Sox2, Cxcr4, c-Myc and β-catenin were detected by R using TCGA data. The mRNA expression of CD44, β-catenin, Cxcr4 and c-Myc was negatively related to Sestrin2, suggesting when upregulating sestrin2 the other factors might downregulate, which was consistent with our in vitro results (Fig. 4H). The expression of Sestrin2 was negatively related to cancer stemness in TCGA data.

In summary, Sestrin2 inhibits the growth of CRC in vivo by downregulating cancer stemness.

## Discussion

Previous studies have introduced the role of Sestrin2 in CRC. In this study, we further investigated the role of Sestrin2 in CRC and found Sestrin2 inhibits cancer stemness both in vivo and in vitro. Using primary and secondary sphere formation assays to detect the self-renewal of CRC cells, we found that Sestrin2 upregulation inhibited sphere formation of CRC cells. Consistently, ELDA suggested that Sestrin2 inhibited their sphere forming ability. Furthermore, the expression of CD44, a key marker for CRC, and Oct4, Sox2, and Cxcr4, stem cell factors, was also decreased. Overall, our data suggested that Sestrin2 not only reduces self-renewal but also inhibits initial ability of CRCs to form.

Sestrin2 may be a therapeutic target. First, CSCs play a very important role in cancer, including maintenance, self-renewal, division, and tumor development [33]. As tumor propagation initiators, CSCs are considered to be a promising therapeutic target [34]. In addition, in this study, Sestrin2 targeting the Wnt/β-catenin pathway reduced the cancer stemness of CRC cells, and Sestrin2 inhibited the proliferation, migration, and colony formation of CRC cells. Treatment targeting Sestrin2 would affect the major tumor biological characteristics of CRC. Thus, Sestin2 may be a promising therapeutic target for CRC.

EMT and CSCs are two closely related processes in tumor progression and therapeutic resistance [35]. In previous studies, Sestrin2 was shown to inhibit EMT [36, 37]. These results provide evidence that Sestrin2 regulates cancer stemness.

Sestrin2 is an anticancer molecule. In CRC, the decreased expression of Sestrin2 in patients predicts unfavorable outcomes, and Sestrin2 is also an important facilitator of the p53-mediated control of cancer cell growth [11, 38]. Moreover, Sestrin2 has been shown to function as a tumor suppressor in lung cancer, hepatocellular carcinoma, and melanoma [39–41]. Antitumor molecules such as tanshinone IIA, fangchinoline, and nelfinavir work together with Sestrin2 to carry out these functions [42–44]. This study showed that Sestrin2 also has anticancer effects.

The effects of Sestrin2 on the Wnt pathway may occur via the AKT signaling pathway. Usually, Sestrin2, known as a p53 target gene, inhibits mTORC1 signaling and the induction of autophagy [45, 46]. Interestingly, our results indicated that Sestrin2 also downregulates the Wnt/β-catenin signaling pathway. The reasons why our results differ from others may be that SENS2 activates AKT and AMPK signaling [39] and PI3K/Akt/Wnt/β-catenin signaling, which controls the levels of EMT-related proteins [37]. Thus, Sestrin2 may affect β-catenin by interacting with upstream factors.

In conclusion, the results of this study showed that LV-Sesrin2 inhibits cancer stemness through the Wnt pathway in HCT-116 and SW620 cells, suggesting that Sesrin2 may be a promising therapeutic target for CRC.

## Supplementary Information

**Additional file 1: Figure S1.** Cell invasion was detected by Transwell assay. The cell number was counted by ImageJ and normalized to the LV-GFP group (right panel) (*P=0.05; Mann–Whitney test; lines showed medians).

**Additional file 2: Figure S2.** **(A) **The relative protein expression of Sestrin2, Sox2, Oct4, Cxcr4, and CD44 in the LV-GFP and LV-Sestrin2 groups of HCT-116 and SW620 cells (*P=0.05; Mann–Whitney test; lines showed medians). **(B) **Representative western blot images of the effect of LV-Sestrin2 on the expression levels of Sestrin2, Sox2, Oct4, and Cxcr4 in HCT-116 and SW620 sphere cells. β-actin was used as a loading control. The relative protein expression is on the right (*P=0.05; Mann–Whitney test; lines showed medians). **(C)** western blot images of the effect of LV-Sestrin2 on the expression levels of E-cadherin. The relative protein expression is on the right (*P=0.05; Mann–Whitney test; lines showed medians).

**Additional file 3: Figure S3. **Western blot images of the effect of LV-Sestrin2 with or without 48 h of treatment with scavengers of ROS, sodium pyruvate and carboxy-PTIO, on the expression levels of CD44 in HCT-116 and SW620 cells. β-actin was used as a loading control.

**Additional file 4: Figure S4. (A) **The mouse body weights were measured. (B) The relative protein expression of Sestrin2, CD44, Sox2, c-Myc, and β-catenin in mouse tumors (*P=0.05; Mann–Whitney test; lines showed medians).

**Additional file 5: Table S1. **The qPCR primer sequences used in the current study.

## Data Availability

The data and materials can be obtained from the first author and corresponding author.
