# Supplementary figures and images for: Sestrin2 reduces cancer stemness via Wnt/β-catenin signaling in colorectal cancer
Source: Cancer Cell Int. 2022 Feb 11;22:75. doi: 10.1186/s12935-022-02498-x (PMC8840770; doi:10.1186/s12935-022-02498-x)

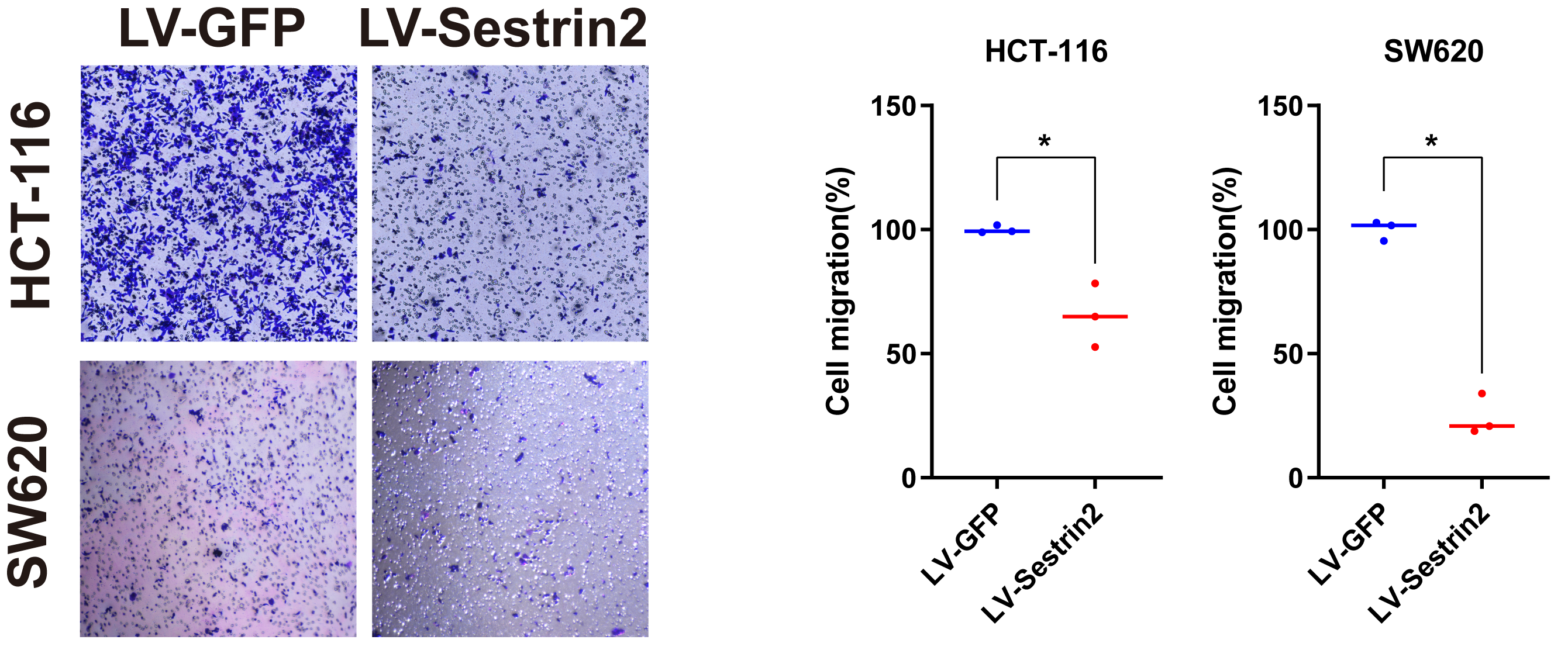

Supplement: Supplementary file 1 — Additional file 1: Figure S1. Cell invasion was detected by Transwell assay. The cell number was counted by ImageJ and normalized to the LV-GFP group (right panel) (*P=0.05; Mann–Whitney test; lines showed medians). [file 12935_2022_2498_MOESM1_ESM.tif]

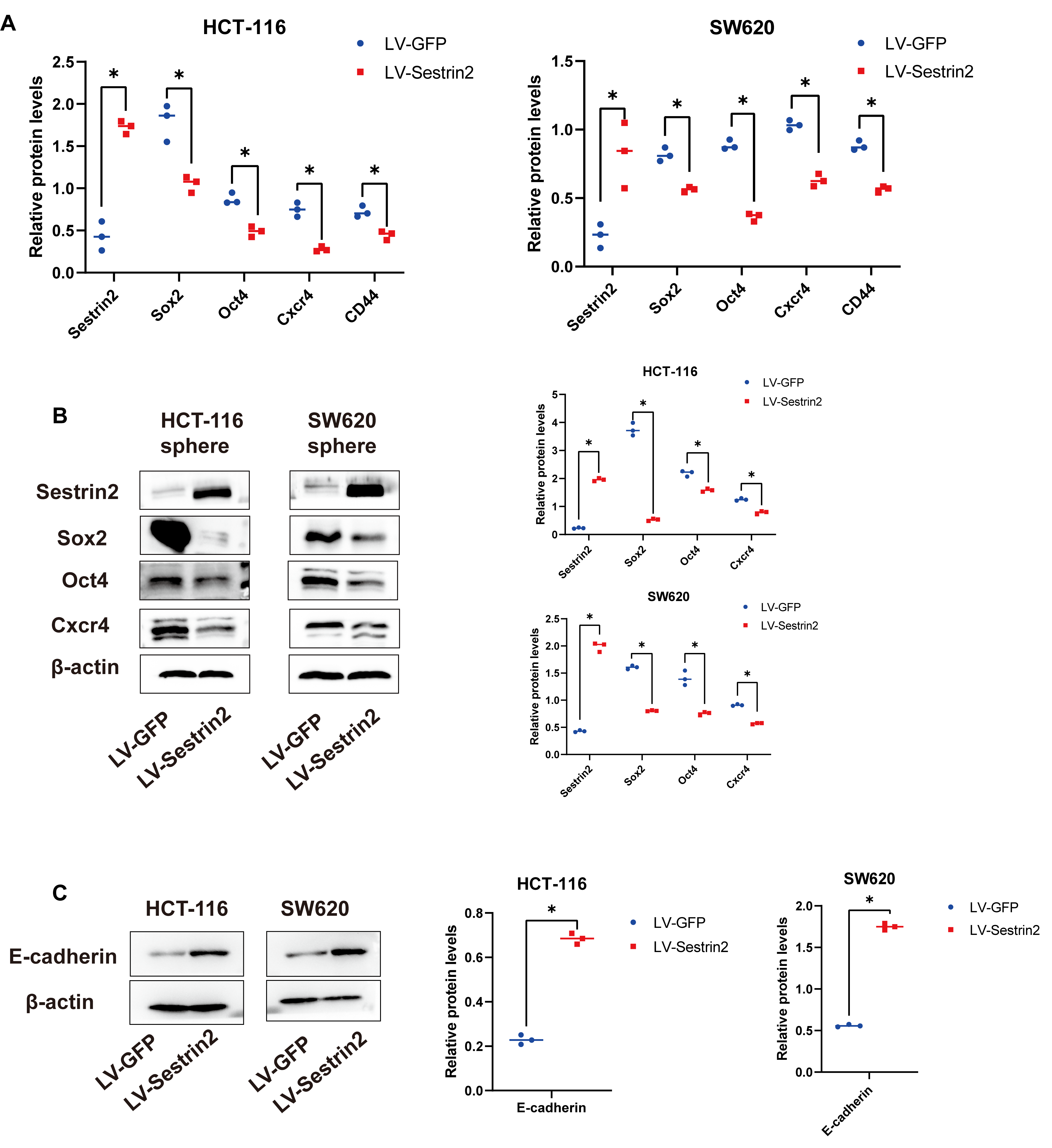

Supplement: Supplementary file 2 — Additional file 2: Figure S2. (A) The relative protein expression of Sestrin2, Sox2, Oct4, Cxcr4, and CD44 in the LV-GFP and LV-Sestrin2 groups of HCT-116 and SW620 cells (*P=0.05; Mann–Whitney test; lines showed medians). (B) Representative western blot images of the effect of LV-Sestrin2 on the expression levels of Sestrin2, Sox2, Oct4, and Cxcr4 in HCT-116 and SW620 sphere cells. β-actin was used as a loading control. The relative protein expression is on the right (*P=0.05; Mann–Whitney test; lines showed medians). (C) western blot images of the effect of LV-Sestrin2 on the expression levels of E-cadherin. The relative protein expression is on the right (*P=0.05; Mann–Whitney test; lines showed medians). [file 12935_2022_2498_MOESM2_ESM.tif]

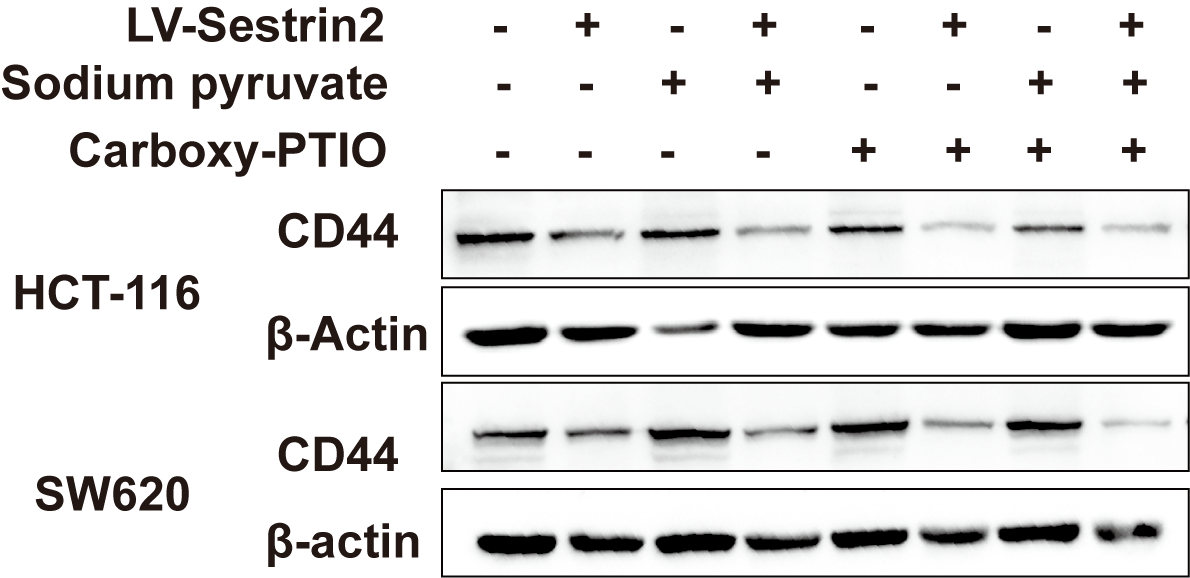

Supplement: Supplementary file 3 — Additional file 3: Figure S3. Western blot images of the effect of LV-Sestrin2 with or without 48 h of treatment with scavengers of ROS, sodium pyruvate and carboxy-PTIO, on the expression levels of CD44 in HCT-116 and SW620 cells. β-actin was used as a loading control. [file 12935_2022_2498_MOESM3_ESM.tif]

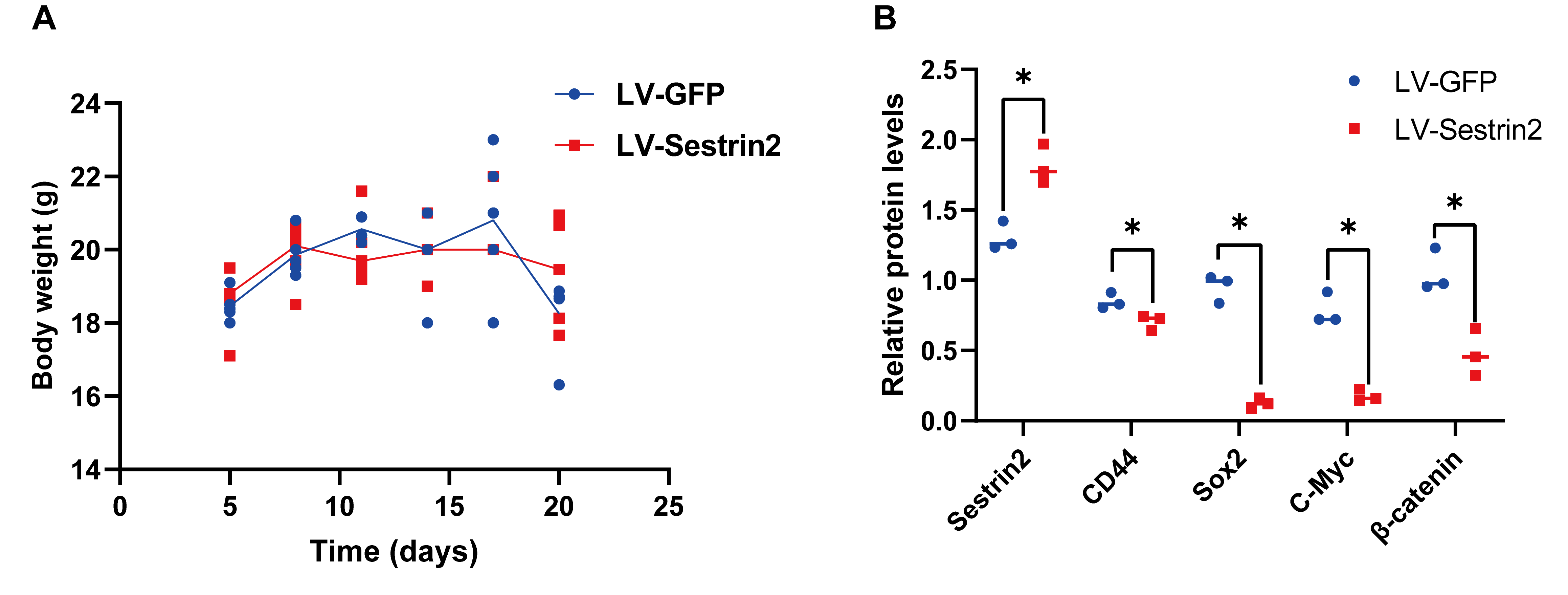

Supplement: Supplementary file 4 — Additional file 4: Figure S4. (A) The mouse body weights were measured. (B) The relative protein expression of Sestrin2, CD44, Sox2, c-Myc, and β-catenin in mouse tumors (*P=0.05; Mann–Whitney test; lines showed medians). [file 12935_2022_2498_MOESM4_ESM.tif]
