# Supplementary material for: Sestrin2 reduces cancer stemness via Wnt/β-catenin signaling in colorectal cancer
Source: Cancer Cell Int. 2022 Feb 11;22:75. doi: 10.1186/s12935-022-02498-x (PMC8840770; doi:10.1186/s12935-022-02498-x)
Supplement: Supplementary file 5 — Additional file 5: Table S1. The qPCR primer sequences used in the current study. [file 12935_2022_2498_MOESM5_ESM.docx]

**Additional file 5: Table S1**

The qPCR primer sequences used in the current study

| Gene | NCBI  accession number | Primer sequences |
| --- | --- | --- |
| Sestrin2 | NM_031459 | Forward: CAACCTGCCAACAGCAAG  Reverse: CCTGGAAGCAACCCACTTA |
| CD44 | NM_001202555 | Forward: GTGCCTCTTGTTTTCCCA  Reverse: GTGGCTTGTTGCTTTTCAG |
| Cxcr4 | NM_003467 | Forward: ATCATCAAGCAAGGGTGTG  Reverse: GGCTCCAAGGAAAGCATAG |
| Oct4 | NM_001285986 | Forward: TGTCTCCGTCACCACTCTG  Reverse: CACCCTTTGTGTTCCCAAT |
| Sox2 | NM_003106 | Forward: CATCCACACTCACGCAAA  Reverse: CTCCCCAGGTTTTCTCTGT |
| β-Actin | NM_001101 | Forward: CTATCACCTCCCCTGTGTG  Reverse: TCCCTTGCCCTCCTAAA |
